# Quantile-dependent expressivity of plasma adiponectin concentrations may explain its sex-specific heritability, gene-environment interactions, and genotype-specific response to postprandial lipemia

**DOI:** 10.7717/peerj.10099

**Published:** 2020-10-14

**Authors:** Paul T. Williams

**Affiliations:** Molecular Biophysics & Integrated Bioimaging, Lawrence Berkeley National Laboratory, Berkeley, CA, United States of America

**Keywords:** Adiponectin, Genetics, Sex, Weight loss, Diabetes, Insulin resistance, ADIPOQ, Postprandial, GWAS

## Abstract

**Background:**

“Quantile-dependent expressivity” occurs when the effect size of a genetic variant depends upon whether the phenotype (e.g. adiponectin) is high or low relative to its distribution. We have previously shown that the heritability (*h^2^*) of adiposity, lipoproteins, postprandial lipemia, pulmonary function, and coffee and alcohol consumption are quantile-specific. Whether adiponectin heritability is quantile specific remains to be determined.

**Methods:**

Plasma adiponectin concentrations from 4,182 offspring-parent pairs and 1,662 sibships from the Framingham Heart Study were analyzed. Quantile-specific heritability from offspring-parent (*β*_OP_,*h^2^* = 2*β*_OP_/(1 + r_spouse_)) and full-sib regression slopes (*β*_FS_, *h^2^* = {(1 + 8r_spouse_*β*_FS_)^0.05^-1}/(2r_spouse_)) were robustly estimated by quantile regression with nonparametric significance assigned from 1,000 bootstrap samples.

**Results:**

Quantile-specific *h^2^* (± SE) increased with increasing percentiles of the offspring’s age- and sex-adjusted adiponectin distribution when estimated from *β*_OP_ (*P*_trend_ = 2.2 × 10^−6^): 0.30 ± 0.03 at the 10th, 0.33 ± 0.04 at the 25th, 0.43 ± 0.04 at the 50th, 0.55 ± 0.05 at the 75th, and 0.57 ± 0.08 at the 90th percentile, and when estimated from *β*_FS_ (*P*_trend_ = 7.6 × 10^−7^): 0.42 ± 0.03 at the 10th, 0.44 ± 0.04 at the 25th, 0.56 ± 0.05 at the 50th, 0.73 ± 0.08 at the 75th, and 0.79 ± 0.11 at the 90th percentile. Consistent with quantile-dependent expressivity, adiponectin’s: (1) heritability was greater in women in accordance with their higher adiponection concentrations; (2) relationships to *ADIPOQ* polymorphisms were modified by adiposity in accordance with its adiponectin-lowering effect; (3) response to rosiglitazone was predicted by the 45T> G *ADIPOQ* polymorphism; (4) difference by *ADIPOQ* haplotypes increased linearly with increasing postprandial adiponectin concentrations.

**Conclusion:**

Adiponectin heritability is quantile dependent, which may explain sex-specific heritability, gene-environment and gene-drug interactions, and postprandial response by haplotypes.

## Introduction

Adiponectin is a 30 kDa circulating adipocyte-derived protein that is a potent insulin sensitizer that regulates energy homeostasis and glucose tolerance in muscle and liver (Swarbrick & Havel, 2008). Low adiponectin concentrations are associated with insulin resistance, type 2 diabetes mellitus (T2DM), coronary artery disease, lipodystrophy, nonalcoholic hepatic steatosis, and essential hypertension, and they precede the development of insulin resistance and myocardial infarction (Swarbrick & Havel, 2008). Meta-analysis showed that low plasma adiponectin concentrations predicted increased T2DM risk in 14,598 subjects from 13 prospective studies (Li et al., 2009). Paradoxically, prospective studies also find that high adiponectin concentrations is a risk factor for all-cause and cardiovascular mortality (Menzaghi & Trischitta, 2018).

Twenty published estimates of adiponectin heritability show its plasma concentrations to be highly heritable (i.e., *h*^2^ = 0.39 (Lindsay et al., 2003; Liu et al., 2008), 0.42 (Hicks et al., 2007; Comuzzie et al., 2001), 0.47 (Vaughan et al., 2015) 0.48 (Chuang et al., 2004; Dosaev, Prakash & Livshits, 2014), 0.55 (Pollin et al., 2005; Henneman et al., 2010), 0.58 (Menzaghi et al., 2010), 0.62 (Al-Daghri et al., 2011), 0.64 (Guo et al., 2006), 0.67 (Ling, Waterworth et al., 2009), 0.68 (Ling, Waterworth et al., 2009), 0.70 (Chuang et al., 2004; Vasseur et al., 2002), 0.71 (Guo et al., 2006), 0.79 (Menzaghi et al., 2010), 0.88 (Cesari et al., 2007), 0.93 (Butte et al., 2005)). None report any difference in heritability between sexes. All but two studies (Hicks et al., 2007; Comuzzie et al., 2001) used adiponectin concentrations that were logarithmically (Lindsay et al., 2003; Liu et al., 2008; Chuang et al., 2004; Dosaev, Prakash & Livshits, 2014; Pollin et al., 2005; Henneman et al., 2010; Menzaghi et al., 2010; Al-Daghri et al., 2011; Guo et al., 2006; Vasseur et al., 2002; Cesari et al., 2007; Butte et al., 2005) or cube-root transformed (Vaughan et al., 2015; Ling, Waterworth et al., 2009). The variation in heritability estimates across reports is likely the result of small sample size, different statistical methodologies, differences between twin- and pedigree-based estimates, and population heterogeneity.

“Quantile-dependent expressivity” is said to occur when the phenotypic expression of a gene depends upon the percentile of the phenotype, i.e., whether the trait (e.g., adiponectin) is high or low relative to its distribution (Williams, 2012). This is in contrast to the traditional estimate of a genetic effect size that is assumed to be constant across all population percentiles. Quantile-dependent expressivity has been demonstrated for adiposity (Williams, 2012; Williams, 2020c), lipoproteins (Williams, 2012; Williams, 2020e; Williams, 2020a), pulmonary function (Williams, 2020g), coffee intake (Williams, 2020d), and alcohol intake (Williams, 2020f). Moreover, the genetic effect sizes of single nucleotide polymorphisms (SNPs) affecting triglycerides have been shown to increase and decrease within individuals in accordance with increasing and decreasing postprandial triglyceride concentrations, consistent with quantile-dependent expressivity (Williams, 2020b).

An important consequence of quantile-dependent expressivity is that the selection of subjects for characteristics that distinguish high vs. low phenotypes can yield different genetic effects (Williams, 2012; Williams, 2020e). Adiponectin concentrations are greater in women than men (Liu et al., 2008; Hicks et al., 2007; Comuzzie et al., 2001; Vaughan et al., 2015; Chuang et al., 2004; Dosaev, Prakash & Livshits, 2014; Henneman et al., 2010; Guo et al., 2006; Ling, Waterworth et al., 2009; Vasseur et al., 2002; Cesari et al., 2007; Berra et al., 2006), increase with rosiglitazone treatment (Kang et al., 2005), increase during postprandial lipemia (Musso et al., 2008), and decrease with adiposity (De Luis et al., 2020; De Luis et al., 2018; De Luis et al., 2019; Corbi et al., 2019; Divella et al., 2017; Garcia-Garcia et al., 2014; Berthier et al., 2005; Aller et al., 2019). It remains to be determined whether the heritability of adiponectin concentrations is quantile-dependent, and whether this produces significant heritability differences by sex, genotype-specific increases during rosiglitazone treatment or postprandial lipemia, and gene-environment interactions by adiposity level.

We therefore used nonparametric quantile regression (Koenker & Hallock, 2001; Gould, 1992) to test whether untransformed adiponectin concentrations exhibit quantile-dependent heritability in the narrow-sense (*h*^2^) as estimated from offspring-parent (*β*_OP_) and full-sib (*β*_FS_) regression slopes (Falconer & Mackay, 1996) in a large population (Framingham Heart Study Dawber, Meadors & Moore, 1951; Kannel et al., 1979; Splansky et al., 2007). Untransformed concentrations were used because quantile regression does not require normality, and no biological justification has been given for its logarithmic transformation. Heritability was studied because between 5% and 9% of the variation in adiponectin is accounted for by variants within the gene encoding adiponectin (ADIPOQ) and other loci (Dastani et al., 2012; Heid et al., 2010). However, because heritability lacks the specificity of directly measured genotypes, we also examined published studies that measured genetic variants directly from the perspective of quantile-dependent expressivity to establish external validity and generalizability.

## Methods

The methods have been described previously (Williams, 2020c; Williams, 2020e; Williams, 2020a; Williams, 2020g), but are repeated here for completeness. The data were obtained from the National Institutes of Health FRAMCOHORT, GEN3, FRAMOFFSPRING Research Materials obtained from the National Heart Lung and Blood Institute (NHLBI) Biologic Specimen and Data Repository Information Coordinating Center. The Original Framingham cohort consisted of men and women between the ages of 30 and 62 from the town of Framingham, Massachusetts (Dawber, Meadors & Moore, 1951). The Offspring (generation 2) Cohort consisted of 5,124 adult children of the original participants and their spouses who were first examined between 1971 and 1975, re-examined eight years later, and then every three to four years thereafter (Kannel et al., 1979). Children of the Offspring Cohort were recruited to form the Third Generation Cohort (Splansky et al., 2007). Subjects were at least 16 years of age and not self-identified as nonwhite or Hispanic. Adiponectin concentrations were measured on stored blood samples frozen at −80 °C from examination 7 of the Framingham Offspring Cohort and examination 1 of the Framingham Third Generation Cohort by ELISA (R&D Systems) with an average interassay coefficients of variation <5% (Zachariah et al., 2017). The statistical analyses were approved by Lawrence Berkeley National Laboratory Human Subjects Committee (HSC) for protocol “Gene-environment interaction vs. quantile-dependent penetrance of established SNPs (107H021)” LBNL holds Office of Human Research Protections Federal wide Assurance number FWA 00006253. Approval number: 107H021-13MR20. The original surveys were conducted under the direction of the Framingham Heart Study human use committee guidelines, with signed informed consent from all participants or parent and/or legal guardian if <18 years of age.

### Statistics

Age and sex adjustment was performed separately for each examination of the Offspring and Third Generation Cohorts using standard least-squares regression with the following independent variables: female (0,1), age, age^2^, female x age, and female × age^2^. Individual subject values were taken as the average of the residuals over all available examinations. Offspring-parent correlations and regression slopes were computed by assigning a weight of one-half to the child-father and one-half to the child-mother pair (if both parents available), and assigning a weight of one to the child-parent pair if only one parent was available. Offspring-midparental correlations and regression slopes were computed by comparing each child’s age and sex-adjusted value to the average of the age and sex-adjusted parental values in those families having both parents. Full-sibling correlations were obtained by constructing all possible pairs using double entry (Karlin, Cameron & Williams, 1981). Unadjusted quantile regression analysis means an unadjusted dependent variable (e.g., offspring, sib) was compared to the age and sex-adjusted independent variables (i.e., parent, other sibs). The number of degrees of freedom for the standard error was adjusted to Σk_*i*_-2 for offspring-parent and midparental regression slopes and correlations, and Σ(k_*i*_-1) for sibship correlations and regression slopes, where k_*i*_ is the number of offspring in family *i* and the summation is taken over all *i*, *i* = 1,…, N nuclear families (Karlin, Cameron & Williams, 1981). Slopes are presented ±SE.

**Figure 1:**
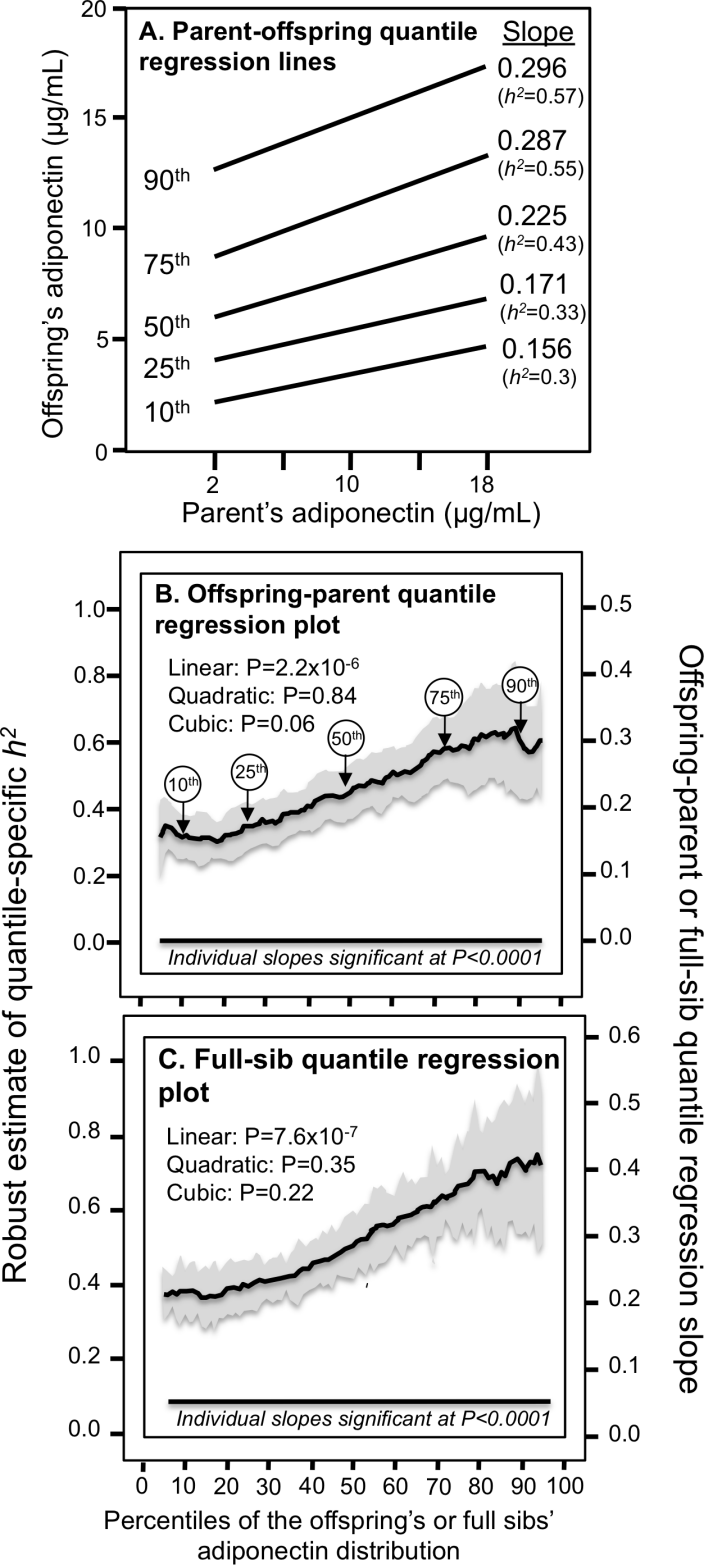
Offspring-parent and full-sib quantile regression slopes. (A) Offspring-parent regression slopes (*β*_*OP*_) for selected quantiles of the offspring’s adiponectin concentrations from 4,182 offspring-parent pairs, with corresponding estimates of heritability (*h*^2^ = 2*β*_*OP*_∕(1 + *r*_spouse_), where the correlation between spouses was *r*_spouse_ = 0.04. The slopes became progressively greater (i.e., steeper) with increasing quantiles of the adiponectin distribution. (B) The selected quantile-specific regression slopes were included with those of other quantiles to create the quantile-specific heritability function in the lower panel. Significance of the linear, quadratic and cubic trends and the 95% confidence intervals (shaded region) determined by 1,000 bootstrap samples. (C) Quantile-specific full-sib regression slopes (*β*_*FS*_) from 4,587 siblings in 1,662 sibships, with corresponding estimates of heritability as estimated by *h*^2^ = (8*r*_spouse_*β*_*FS*_ + 1)^0.5^ − 1∕(2*r*_spouse_). 95% confidence intervals (shaded region) determined by 1,000 bootstrap samples.

Simultaneous quantile regression is a well-developed statistical procedure (Koenker & Hallock, 2001) that estimates the regression coefficients for multiple quantiles using linear programming to minimize the sum of asymmetrically weighted absolute residuals, and bootstrap resampling to estimate their corresponding variances and covariances (Gould, 1992). Simultaneous quantile regression was performed using the “sqreg” command of Stata (version. 11, StataCorp, College Station, TX) with one thousand bootstrap samples drawn to estimate the variance–covariance matrix for the 91 quantile regression coefficients between the 5th and 95th percentiles, and the post-estimation procedures (test and lincom) to test linear combinations of the slopes after estimation with Σk_i_-2 degrees of freedom for offspring-parent regression slopes and Σ(k_i_-1) degrees of freedom for sibship regression slopes. Quantile-specific expressivity was assessed by: (1) estimating quantile-specific *β*-coefficient for the 5th, 6th,..., 95th percentiles of the sample distribution using simultaneous quantile regression (Fig. 1, the <5th and >95th percentiles ignored because they were thought to be less stable); (2) plotting the quantile-specific *β* coefficients vs. the percentile of the trait distribution; and (3) testing whether the resulting graph is constant, or changes as a linear, quadratic, or cubic function of the percentile of the trait distribution using orthogonal polynomials (Winer, Brown & Michels, 1991). Heritability in the narrow sense (*h*^2^) was estimated as *h*^2^ = 2*β*_OP_/(1+r_spouse_) from offspring-parent regression slopes (*β*_OP_), *h*^2^ = *β*_OM_ from the offspring midparental slope (*β*_OM_), and *h*^2^ = {(1+8r_spouse_ *β*_FS_)^0.5^-1}/2r_spouse_ from full-sibs regression slopes (*β*_FS_) where r_spouse_ is the spouse correlation (Falconer & Mackay, 1996) “Quantile-specific heritability” refers to the heritability statistic (*h*^2^), whereas “quantile-dependent expressivity” is the biological phenomenon of the trait expression being quantile-dependent.

When *β*_OP_ for male and female offspring are included on the same graph, their quantile-specific functions compares their heritabilities at the corresponding percentiles of their separate distribution (e.g., the slope at the 50th percentile of the daughters’ distribution vs. the slope at the 50th percentile of the sons’ distribution). However, the adiponectin concentration at the 50th percentile of the daughters’ distribution will be greater then the 50th percentile of the sons’ distribution. Quantile-specific expressivity postulates that the genetic effects depend upon the adiponectin concentration. Therefore, additional displays were created using Q-Q plots (Wilk & Gnanadesikan, 1968) to re-plot the sons’ and daughters’ heritability at the same adiponectin concentrations.

In the discussion, the results from other studies were re-interpreted from the perspective of quantile-dependent expressivity using the genotype-specific mean adiponectin concentrations presented in the original articles or by extracting these values from graphs using the Microsoft Powerpoint formatting palette (version 12.3.6 for Macintosh computers, Microsoft corporation, Redmond WA) as previously described (Williams, 2020b).

### Data availability

The data are not being published in accordance with the data use agreement between the NIH National Heart Lung, and Blood Institute and Lawrence Berkeley National Laboratory. However, the data that support the findings of this study are available from NIH National Heart Lung, and Blood Institute Biologic Specimen and Data Repository Information Coordinating Center directly through the website https://biolincc.nhlbi.nih.gov/my/submitted/request/ (National Heart, Lung, and Blood Institute, 2020b). Restrictions apply to the availability of these data, which were used under license for this study. Those wishing a copy of the data set should contact the Blood Institute Biologic Specimen and Data Repository Information Coordinating Center at the above website, where they can find information on human use approval and data use agreement requiring signature by an official with signing authority for their institute. The public summary-level phenotype data may be browsed at the dbGaP study home page (Genotypes and Phenotypes (dbGaP), 2020a).

## Results

### Traditional estimates of familial concordance and heritability

The sample characteristics displayed in Table 1 show average adiponectin were significantly higher in women than men. BMI was negatively correlated with adiponectin concentrations (*r* =  − 0.31) when age and sex adjusted. Spouse correlation for adjusted adiponectin concentrations was weak (r_spouse_ = 0.04). The offspring-parent regression slope for adjusted adiponectin concentrations (*β*_OP_ ± SE: 0.22 ± 0.01), computed from 1718 offspring with one parent and 1232 offspring with two parents, corresponds to a heritability (*h*^2^) of 0.43 ± 0.03, the same as when estimated from *β*_OM_ (*β*_OM_=0.43 ± 0.03). There were 4587 full-sibs in 1662 sibships with age and sex-adjusted adiponectin concentrations, whose full-sib regression slope (*β*_FS_) was 0.29 ± 0.02, which from Falconer’s formula, corresponds to a heritability of *h* ^2^=0.57 ± 0.04.

**Table 1:** Sample characteristics.

	Males		Females	
	Offspring cohort	Third generation cohort	Offspring cohort	Third generation cohort
Age, years	61.21 (9.63)	40.44 (8.62)	60.93 (9.41)	39.91 (8.73)
BMI, kg/m^2^	28.62 (4.62)	27.99 (4.67)	27.43 (5.80)	26.03 (6.11)
Adiponectin, µg/mL	7.45 (6.63)	6.09 (3.82)	12.59 (6.71)	10.97 (5.77)

### Quantile-dependent expressivity

The *β*_OP_‘s (offspring-parent regression slopes) at the 10th, 25th, 50th, 75th, and 90th percentiles of the offspring’s adiponectin distribution are presented in Fig. 1A, along with their corresponding heritability estimates (*h*^2^= 2*β_OP_/(1+r_spouse_)). The slopes get progressively greater with increasing percentiles of the adiponectin distribution. The heritability at the 90th percentile was 0.57, which is 89.6% greater than the heritability at the 10th percentile (P_difference_ = 0.001). The quantile-specific heritability plot of Fig. 1B presents these slopes, along with those of the other percentiles between the 5th and 95th percentiles. They show heritability increased linearly (i.e., slope ± SE: 0.0038 ± 0.0008, P_linear_=2.2 ×10^−6^) with increasing percentiles of the offspring’s distribution. There was no significant evidence of nonlinearity (i.e., P_quadratic_ = 0.84; P_cubic_ = 0.06). Quantile-specific heritability was individually significant (*P* ≤ 7.2 ×10^−7^) for all percentiles of the offspring’s distribution. If the heritability over all quantiles was constant, then the line segments would all be parallel in Figs. 1A, and 1B would show a flat line having zero slope. Figure 1C displays the quantile regression analysis for *h* ^2^ estimated from full-sib regression slopes (*β*_FS_). Each one-percent increase in the adiponectin distribution was associated with a 0.0052 ± 0.001 increase in heritability and a 0.0026 ± 0.0005 increase in the full-sib regression slope (P_linear_=7.6 ×10^−7^).

Significant quantile-dependent expressivity was replicated when 506 sibships from the Offspring Cohort and 1156 sibships from the Third Generation Cohorts were analyzed separately, i.e., *β*_FS_ increased 0.0023 ± 0.0011 in the Offspring Cohort (*P* = 0.04) and 0.0028 ± 0.0006 in the Third Generation Cohort (*P* = 8.0 ×10^−6^) for each one-percent increment in the sibs’ adjusted adiponectin concentrations.

### Male–female differences in heritability

**Figure 2:**
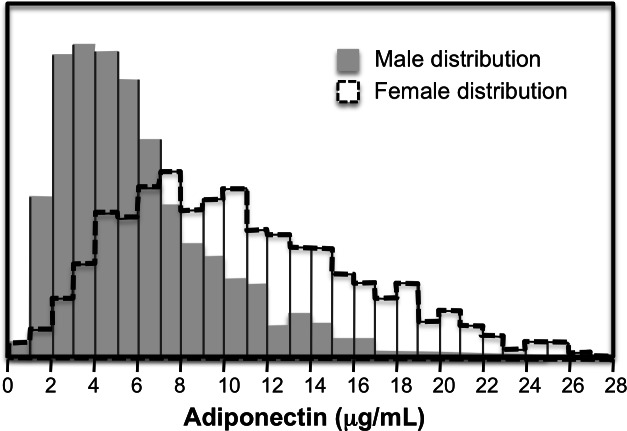
Distribution of fasting adiponectin concentrations in males and females.

**Figure 3:**
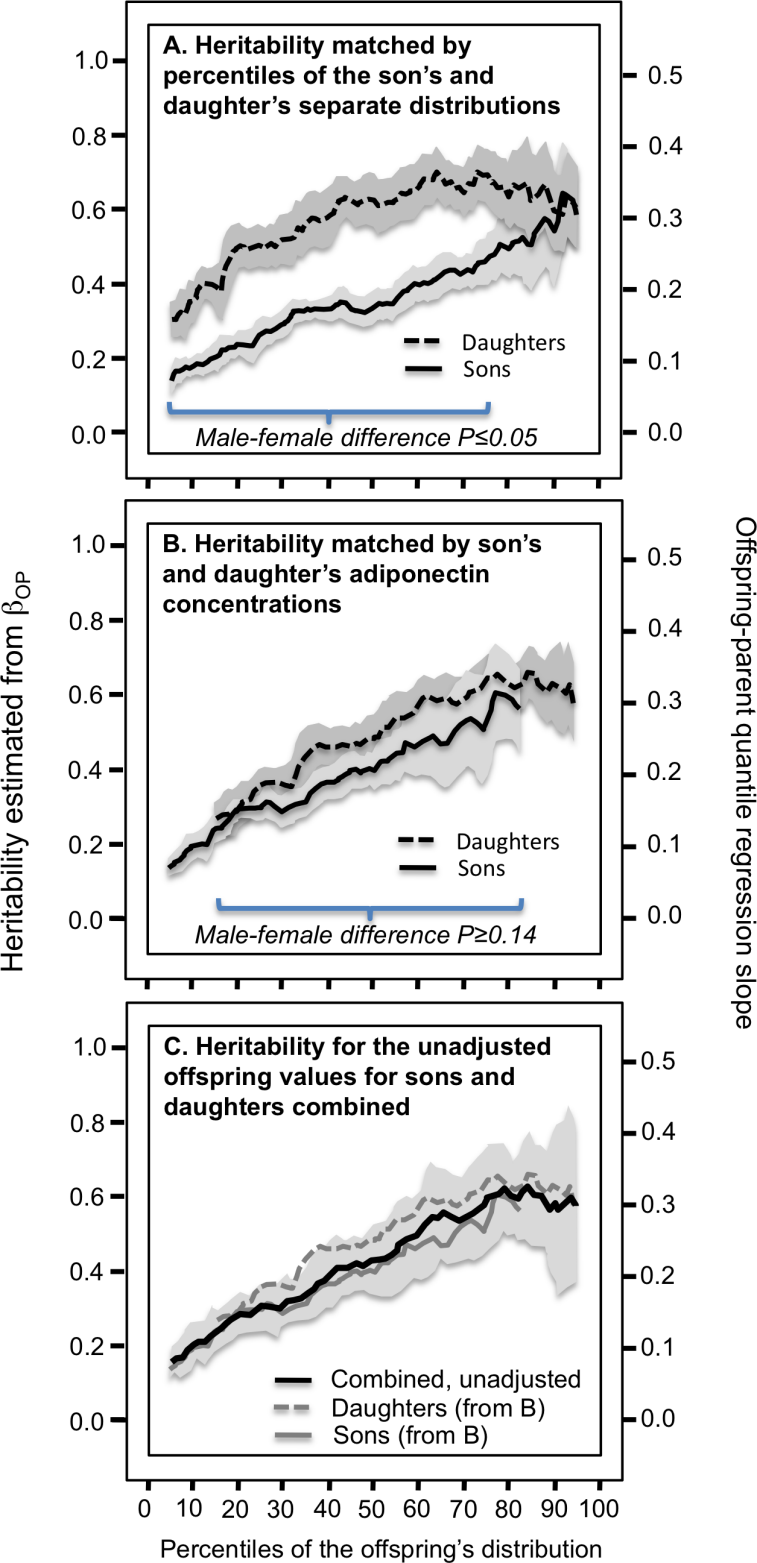
Offspring-parent quantile regression slopes (*β*_OP_) in male and female offspring separately. (A) Offspring-parent regression slopes (*β*_OP_) in male and female offspring separately from age- and sex-adjusted parent-son and parent-daughter pairs, showing their significant difference when the slopes are compared at their corresponding percentiles (the sons’ vs. the daughters’ *β*_OP_ compared at the 5th percentile of separate distributions, the 6th percentile of their separate distributions, …, 95th percentile of their separate distributions). Shaded area designates ±SE; (B) Offspring-parent regression slopes (*β*_OP_) in male and female offspring showing the significant difference is eliminated when compared at their corresponding adiponectin concentrations (the sons’ vs. the daughters’ *β*_OP_ translated using quantile-quantile (Q-Q) plots to the adiponectin concentrations at the 5th percentile of their combined distribution, the 6th percentile of their combined distribution, …, 95th percentile of their combined distribution). Shaded area designates ±SE. (C) Offspring-parent regression slopes for sons and daughters combined without adjustment for sex, showing the unadjusted analysis provides a simpler description of the quantile increase based solely on the percentiles of their unadjusted adiponectin concentrations. Note that the separate curves for sons’ and daughters’ fall fully within the 95% confidence interval (shaded area) for their combined sex-unadjusted analysis.

The preceding analyses showed that adiponectin heritability increased with increasing percentiles of the offspring distribution for the combined sample of male and female age- and sex-adjusted offspring. Figure 2 however, shows that the female adiponectin distribution is shifted towards to the right of the males. Correspondingly, the analyses of Fig. 1B suggest that female heritability should be greater than that of the males. In fact, heritability as classically estimated by standard regression was higher in females than males for adiponectin (0.53 ± 0.05 vs. 0.33 ± 0.03, *P* < 10^−15^) and Fig. 3A shows that the quantile-specific heritability was higher in females than males at each percentile of their respective distribution. Adiponectin heritability was significantly greater in females than males (*P* < 0.05) for each percentile between the 8th and the 77th percentile.

**Figure 4:**
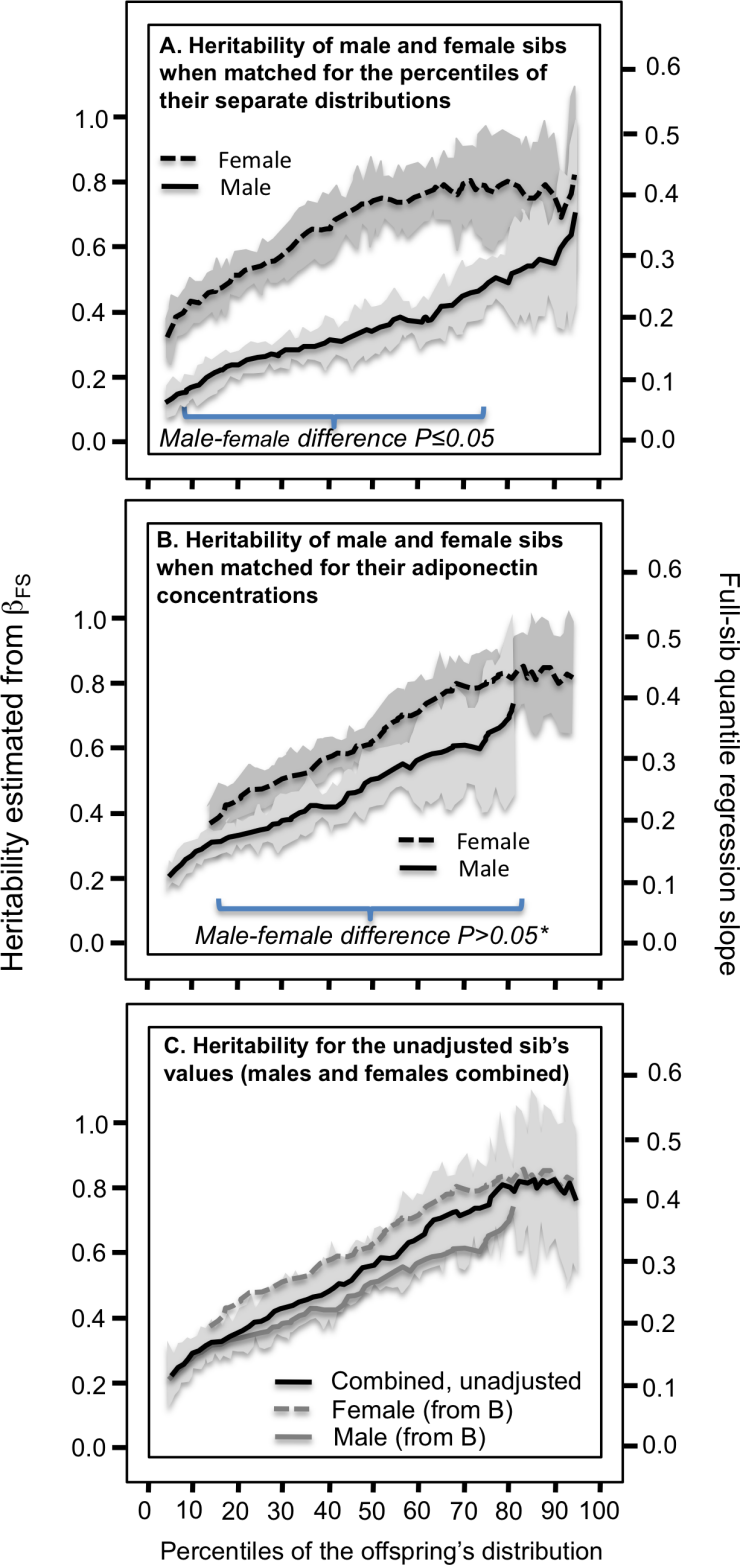
Full-sib quantile regression slopes (*β*_*FS*_) in male and female offspring separately. (A) Analyses showing that the full sib regression slopes (*β*_*FS*_) was greater in female than male siblings when matched by their corresponding percentiles, (B) but not when matched by their corresponding adiponectin concentrations, and (C) that a simpler graph of their combined male and female sibs, unadjusted for sex, includes their separate curves within its 95% confidence interval. See legend to Fig. 3 for details. *exceptions were *P* = 0.05 at the 39th, *P* = 0.04 at the 40th, and *P* = 0.03 at the 42nd percentiles.

From the perspective of quantile-dependent expressivity, the problem with Fig. 3A is that comparing male and female heritability at their 10th percentiles means comparing the male heritability at an unadjusted adiponectin concentration of 2.25 µg/ml with the female heritability at an unadjusted concentration 4.25 µg/ml, comparing their heritability at their 50th percentile means comparing the male heritability at 5.18 µg/ml with the female heritability at 9.98 µg/ml, and comparing their heritability at the 90th percentiles means comparing the male heritability at 11.41 µg/ml with the female heritability at 18.91 µg/ml. Specifically, quantile-dependent expressivity predicts an increase in heritability with increasing adiponectin concentrations. Therefore the male and female heritability graphs were re-plotted to the same adiponectin concentrations in Fig. 3B using quantile–quantile (Q-Q) plots (see methods). This eliminated the significant differences between the male and female heritability plots. Similarly, Figs. 4A and 4B present the analyses for the full-sib estimates of heritability showing substantial differences between the male and female graphs when matched by the percentiles of their corresponding age and sex-adjusted distribution that are eliminated when matched by their corresponding unadjusted adiponectin concentrations. Figs. 3C and 4C show that a simple plot of the unadjusted quantile regression slopes by percentiles of the offspring or sib distribution includes the re-plotted male and females graphs of Figs. 3B and 4B within its 95% confidence interval.

## Discussion

Our analyses suggest that plasma adiponectin concentrations exhibit quantile-dependent expressivity. The finding was replicated using the full-sib regression analyses in the Framingham Offspring Cohort (P_linear_ = 0.04) and the Framingham Third Generation Cohort separately (*P*_linear_=8.0 ×10^−6^). Moreover, the stronger adiponectin heritability in female than male offspring can be largely attributed to quantile-dependent expressivity and the females’ higher concentrations (Figs. 3 and 4). A similar analytic approach was previously used to show that quantile-dependent expressivity explained the larger male than female postprandial triglyceride difference for the *APOA5* –1131 T>C polymorphism (Williams, 2020b). These examples suggest pro forma statistical adjustment for sex may conceal important properties of a trait’s heritability. In fact, the replotted heritability of Figs. 3C and 4C show the unadjusted offspring adiponectin concentrations provided the simplest representation of their quantile-specific heritabilities.

Women have higher adiponectin concentrations due at least in part to the adiponection-lowering effects of testosterone (Berra et al., 2006). Whereas sex-differences in adiponectin concentrations are consistently reported (Liu et al., 2008; Hicks et al., 2007; Comuzzie et al., 2001; Vaughan et al., 2015; Chuang et al., 2004; Dosaev, Prakash & Livshits, 2014; Henneman et al., 2010; Guo et al., 2006; Ling, Waterworth et al., 2009; Vasseur et al., 2002; Cesari et al., 2007), sex-differences in their heritabilities are not. This we attribute to their reliance on statistical procedures that require normally distributed data and logarithmic or other data transformations. These transformations accentuate the slope at lower phenotype values and diminish the slope at higher values. For example, using the Framingham data reported here, the traditional (nonquantile) offspring-parent slope (*β*_OP_ ±SE) for female vs. male offspring was 0.2733 ± 0.0238 vs. 0.1697 ± 0.0171 (P_difference_<10^−15^) for the untransformed data and 0.3221 ± 0.0248 vs. 0.3255 ± 0.0294 for the log-transformed data (P_difference_ = 0.93). The important point is that quantile regression and its bootstrap-derived standard errors do not require a normal distribution (Koenker & Hallock, 2001; Gould, 1992). There is no biological imperative to logarithmically or otherwise transform the data. That is not to say that quantile-regression is invariant to data transformations, which they are not (Fig. S1), but rather the rationale for transformations should ideally be biologically based, not statistically based, and its consequences acknowledged.

All the major genomewide association studies were performed on logarithmic (Richards et al., 2009; Jee et al., 2010; Ling et al., 2009; Gu, 2009) or z-score transformed adiponectin concentrations (Bouatia-Naji et al., 2006). Our results suggest this statistical accommodation may work against the goal of identifying SNPs affecting adiponectin concentrations. Specifically, Fig. 1 suggests that the transformation accentuates the genetic effect at low concentrations (where the genetic effects are weakest) and diminishes the genetic effect at higher values concentrations (where the genetic effects are strongest). Our previous analyses (Williams, 2012; Williams, 2020c; Williams, 2020e; Williams, 2020a; Williams, 2020b) suggest this concern is also apropos to lipoproteins and adiposity GWAS.

Important caveats to our analysis of phenotypes in family sets are: (1) heritability lacks the specificity of directly measured genotypes even if it is a more inclusive measure of genetic effects; and (2) Falconer’s formula probably do not adequately address the true complexity of the genetics and shared environment affecting adiponectin concentrations. These concerns can be partly addressed by re-analyzing published studies that measured genetic variants directly from the perspective of quantile-dependent expressivity. They include multiple examples where the paper’s original interpretation from the perspective of precision medicine or gene-environment interactions might be more simply explained by a single underlying phenomenon: quantile-dependent expressivity. Results are presented in their reported units.

### Pharmacogenetics

There is an important distinction between quantile-dependence and pharmacogenetics. Pharmacogenetics attempts to use genetic markers that identify patients most likely to benefit from specific treatments to individualize drug prescriptions. Quantile-dependent expressivity postulates that drugs alter the phenotype (e.g., increase adiponectin concentrations), which in turn alters the expressivity of genetic variants. More simply stated, genetic markers merely track the increase in heritability with increasing adiponectin concentrations.

**Figure 5:**
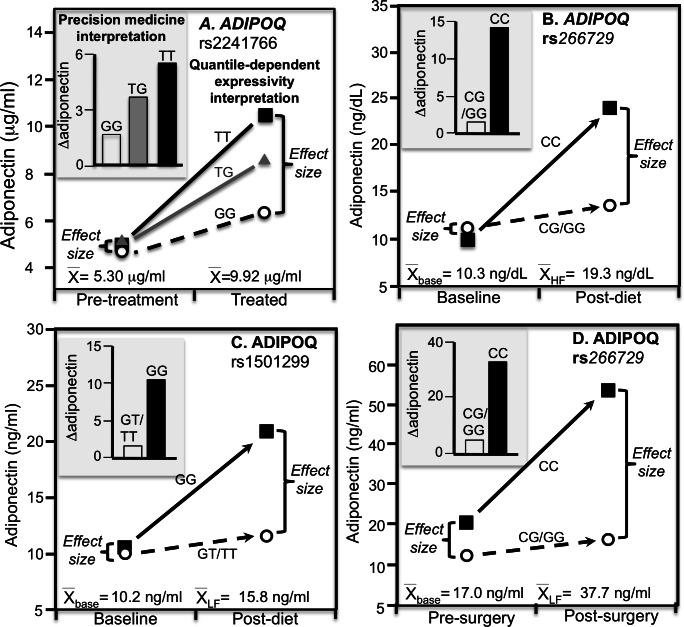
Precision medicine perspective of *ADIPOQ* genotype-specific adiponectin differences (histogram inserts) vs. quantile-dependent expressivity perspective (line graphs). Precision medicine perspective of *ADIPOQ* genotype-specific adiponectin differences (histogram inserts) vs. quantile-dependent expressivity perspective (line graphs showing larger genetic effect size when average adiponectin concentrations were high) for: (A) Kang et al.’s 2005 report  (Kang et al., 2005) on the effect of 12-weeks 4 mg/day of rosiglitazone treatment in 25 GG rs2241766 homozygotes and 86 T-allele carriers with T2DM; (B) de Luis et al.’s 2020 report (De Luis et al., 2020) on the pooled effect of switching from a basal to a 27%- or 38%-fat hypocaloric diet in 169 rs266729 CC homozygotes and 114 G-allele carriers; (C) Aller et al.’s 2019 report (Aller et al., 2019) on the pooled effect of switching from a basal to a standard or high-protein extreme hypocaloric diet in 122 rs1501299 GG homozygotes than 147 T-allele carriers; (D) De Luis et al’s 2018 report (De Luis et al., 2018) on the effect of 41.9 kg weight loss from biliopancreatic diversion surgery in 84 rs266729 CC homozygote and 65 G-allele carriers who were morbidly obesity. The histograms and line graphs were derived from the mean concentrations presented in the original manuscripts.

For example, rosiglitazone is a thiazolidinedione derivate that increases serum adiponectin concentration by increasing adiponectin transcription (Kang et al., 2005). Kang et al. (2005) reported significantly smaller increases in adiponectin concentrations in GG homozygotes of the at position 45 (rs2241766) of the ADIPOQ gene than carriers of the T allele after 166 T2DM’s received 4 mg/day of rosiglitazone for 12 weeks (*P* < 0.003, Fig. 5A histogram). Heterozygotes had an intermediate response. Alternatively, from the perspective of quantile-dependent expressivity (Fig. 5A line graph) there were substantially greater differences in adiponectin concentrations between genotypes at the end of treatment than at baseline (TT minus GG difference: 4.12 ± 1.30 vs. 0.27 ± 0.79 µg/ml) in accordance with the significantly higher mean adiponectin concentrations after treatment than before (9.92 ±0.53 vs. 5.30 ± 0.37 µg/ml, *P* < 0.001).

### Gene-environment interactions

**Figure 6:**
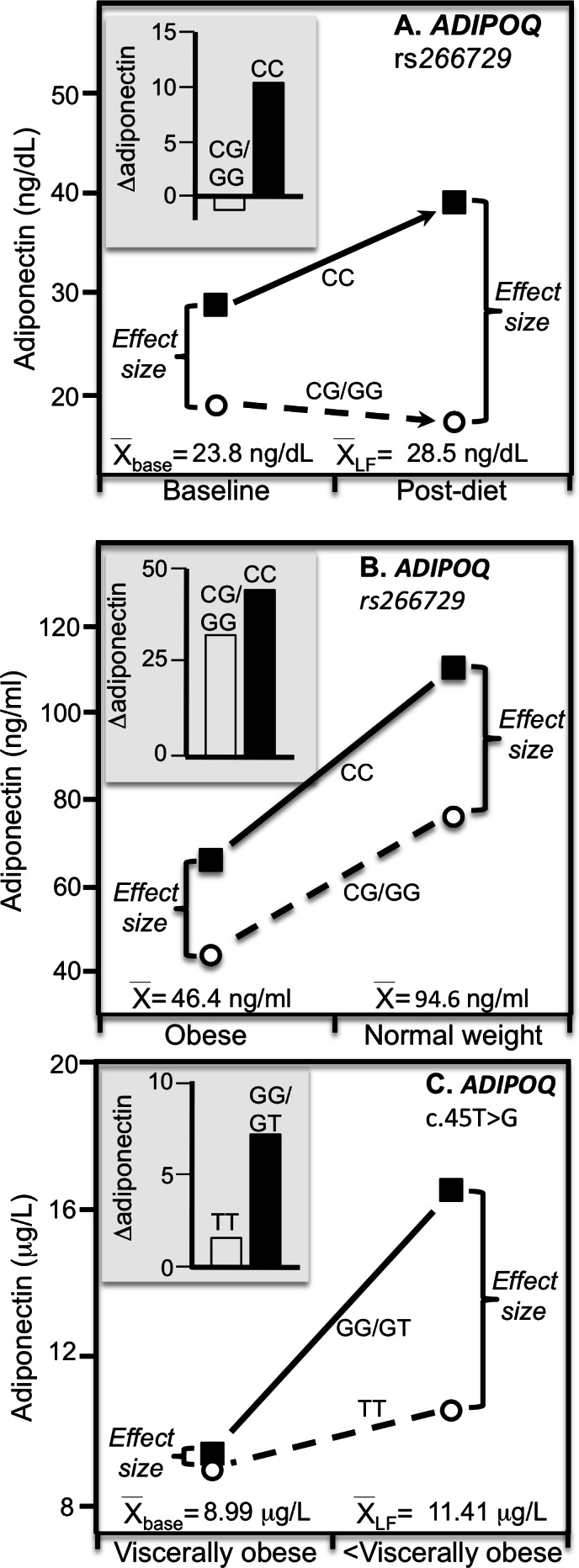
Precision medicine perspective of *ADIPOQ* genotype-specific adiponectin differences (histogram inserts) vs. quantile-dependent expressivity perspective (line graphs). Precision medicine perspective of *ADIPOQ* genotype-specific adiponectin differences (histogram inserts) vs. quantile-dependent expressivity perspective (line graph showing larger genetic effect size when average adiponectin concentrations were high) for: (A) de Luis et al. 2019 report  (De Luis et al., 2019) on a 3-month Mediterranean-type hypocaloric diet in 48 rs266729 CC homozygotes and 45 G-allele carriers; (B) Divella et al. 2017 report (Divella et al., 2017) on the cross-sectional difference between being obese and nonobese in 30 rs266729 CC homozygotes and 73 G-allele carriers with colon cancer; and (C) Berthier et al. (Berthier et al., 2005) 2005 report of the cross-sectional difference between high and low visceral adiposity (computed tomography ≥ 130 vs. <130 cm ^2^) in 26 rs2241766 TT-homozygotes vs. 117 male G-allele carriers. The histograms and line graphs were derived from the mean concentrations presented in the original manuscripts.

There are multiple reports of adiposity modulating genetic influences on adiponectin concentrations, or equivalently, that these polymorphisms modulated the effects of adiposity on adiponectin concentrations (Figs. 5B–5D and Figs. 6A–6C histograms). These include the rs266729 (–11,377C>G) polymorphism located in the proximal promoter region of the ADIPOQ gene and which functionally regulates adiponectin promoter activity and adiponectin levels (Gu, 2009; Bouatia-Naji et al., 2006), rs1501299 (+276T>G) in ADIPOQ’s intron 2, and the aforementioned rs2241766 in ADIPOQ’s exon 2.

De Luis et al. (2020) reported significantly greater increases in adiponectin concentration in CC homozygotes than G-carriers of the ADIPOQ rs266729 gene polymorphism when participants switched from a basal diet to either a 27% low-fat hypocaloric (CC vs. G-carriers: 16.1 ± 2.8 vs. 1.3 ± 1.0 ng/dL, *P* = 0.03) or a 38% high fat hypocaloric diet (10.6 ± 2.0 vs. 1.8 ± 1.0 ng/dL, *P* = 0.01) for three months (Fig. 5B histogram, pooled across diets). Both diets produced significant weight loss: 4.5 ± 0.9 kg on the high-fat and 4.1 ± 0.9 kg on the low-fat diet. Alternatively, on the high-fat diet, the adiponectin difference between genotypes was greater after weight loss (8.3 ± 0.8 ng/dL) when the overall average concentration was higher (16.9 ± 0.4 ng/dL) vis-à-vis before weight loss (−0.5 ± 0.7 ng/dL) when overall average concentration was lower (9.8 ± 0.3 ng/dL). Similarly, on the low-fat diet there was a larger adiponectin difference between genotypes after weight loss (14.0 ± 1.3 ng/dL) at the higher average concentration (21.5 ± 0.8 ng/dL) vis-à-vis before weight loss (−1.8 ± 1.1 ng/dL) at the lower average concentration (10.8 ± 0.7 ng/dL) (De Luis et al., 2020), suggesting that quantile-dependent expressivity may have contributed to the genotype-specific increases (Fig. 5B line graph for the pooled results).

From the same laboratory, Aller et al. (2019) reported greater 9-month increases in adiponectin concentrations in GG homozygotes of the rs1501299 gene than T-allele carriers when switching from their basal diet to one of two severe hypocaloric diets: a standard version and a high-protein low-carbohydrate version. Both diets increased adiponectin significantly in GG homozygotes (standard: 10.9 ng/ml, *P* < 0.05; high-protein: 10.1 ng/ml, *P* < 0.05) but not in carriers of the T allele (standard: 0.6 ng/ml; high-protein: 2.6 ng/ml). Their pooled results are presented in Fig. 5C histogram. However, for both diets average adiponectin concentrations were higher after 9-month weight loss (standard: 15.4 ± 0.5 ng/ml; high-protein: 16.3 ± 0.4 ng/ml) than at baseline (standard: 10.3 ± 0.5 ng/ml; high-protein: 10.1 ± 0.3 ng/ml), and in accordance with quantile-dependent expressivity, the difference between GG and T-allele carriers was greater for the higher average concentrations after weight loss (standard: 11.5 ± 1.0 ng/ml; high-protein: 7.3 ± 0.9 ng/ml) than at the low average concentrations at baseline (standard: 1.2 ± 0.9 ng/ml; high-protein: −0.2 ± 0.6 ng/ml). The line graph of Fig. 5C presents this quantile-dependent interpretation for the pooled sample.

De Luis et al. (2018) also reported that rs266729 CC homozygotes had significantly greater adiponectin increases than G-carriers when 149 morbidly obese patients lost an average of 41.9 kg during the three years following biliopancreatic diversion surgery (Fig. 5D histogram, 33.2 ± 0.4 vs. 4.7 ± 0.2 ng/ml; *P* = 0.01). From the perspective of quantile-dependent expressivity, the genetic effect size between CC homozygotes and G-allele carriers increased as mean adiponectin concentration increased from 17.0 ± 0.4 ng/ml pre-surgery (8.7 ± 0.8 ng/ml difference between genotypes), to 27.1 ± 0.5 ng/ml one-year post surgery (22.5 ± 1.0 ng/ml genotype difference), 31.8 ± 0.4 ng/ml two-years post surgery (29.8 ± 0.9 ng/ml genotype difference), and 37.7 ± 0.5 ng/ml three-years post surgery (37.1 ± 1.1 ng/ml genotype difference).

A third study by De Luis et al. (2019) reported that rs266729 CC homozygotes had significantly greater adiponectin increases than G-carriers (Fig. 6A histogram, 10.4 ±3.1 vs. −1.3 ± 1.0 ng/dL, *P* = 0.01) when 83 obese patients lost an average of 3.5 ± 0.6 kg after a 3-month Mediterranean-type hypocaloric diet. Again, from the perspective of quantile-dependent expressivity, the genetic effect size between CC homozygotes and G-allele carriers increased as mean adiponectin increased from the pre-diet 23.8 ± 0.6 ng/dL average (10.2 ± 1.1 ng/dL genotype difference) to the 28.5 ± 0.4 ng/dL post-diet average (21.9 ± 0.9 ng/dL difference).

Cross-sectionally, Divella et al. (2017) reported that the difference in adiponectin concentration between obese and normal weight colorectal cancer patients was greater in rs266729 CC homozygotes than CG/GG genotypes (44.5 ± 10.4 vs. 32.3 ± 10.1 ng/ml, Fig. 6B histogram). Consistent with quantile-dependent expressivity, the associated line graph shows that the difference between genotypes increased as mean adiponectin concentrations increased from 46.3 ± 4.2 ng/ml in obese (genotype difference 22.1 ± 8.9 ng/ml), 51.8 ± 5.5 ng/ml in overweight (30.6 ± 16.3 ng/ml difference, not displayed), to 94.6 ± 5.7 ng/ml in normal weight patients (34.3 ± 11.5 ng/ml difference).

Garcia-Garcia et al. (2014) concluded that adiponectin levels were modulated by the interaction between BMI and ADIPOQ –11391G/A SNP on the basis of a significant adiponectin difference between GA and GG genotypes in the 1st (1.30 ± 0.66 µg/ml, *P* = 0.03) but not 2nd (0.2 ± 0.29 µg/mL) nor 3rd BMI tertiles (0.2 ± 0.24 µg/ml), consistent with quantile-dependent expressivity given that mean adiponectin concentrations were significantly higher in the 1st (4.20 ± 0.28 µg/ml) than the 2nd (3.09 ± 0.15) or 3rd BMI tertiles (2.30 ± 0.12 µg/ml).

Berthier et al. (2005) reported that visceral adiposity modulated the effect of the rs2241766 *ADIPOQ* gene polymorphism on adiponectin concentrations. Otherwise stated, Fig. 6C histogram (estimated from their figure 1) shows the effect of visceral fat was greater in carriers of the G-allele than TT homozygotes. From the perspective of quantile-dependent expressivity, the genetic effect size was greater in the less-viscerally obese than viscerally obese subjects (6.0 vs. 0.4 µg/L) in accordance with their higher average adiponectin concentrations.

### Sex-specific genetic effects

Quantile-dependent expressivity, in conjunction with the higher average adiponectin concentrations in women than men (6.04 ± 0.10 vs. 4.08 ± 0.10 µg/ml), might explain Riestra et al. (2015) report that ADIPOQ variants rs6444174, rs16861205, rs1403697, and rs7641507 were strongly associated with serum adiponectin concentrations in women but not men.

### Postprandial lipemia

The dependence of genetic effects on mean adiponectin concentrations has also been demonstrated within individuals during their postprandial response. Carriers of the 45TT (rs2241766) and 276GT/TT (rs1501299) *ADIPOQ* haplotype have a higher T2DM and cardiovascular disease risk than noncarriers. As derived from Musso et al.’s report (Musso et al., 2008), Fig. 7 shows that the haplotype’s blunted affects on the postprandial adiponectin concentrations following an oral fat load were linearly related to the average adiponection concentrations at time t (linear regression, 4 df, *P* = 0.0002).

**Figure 7:**
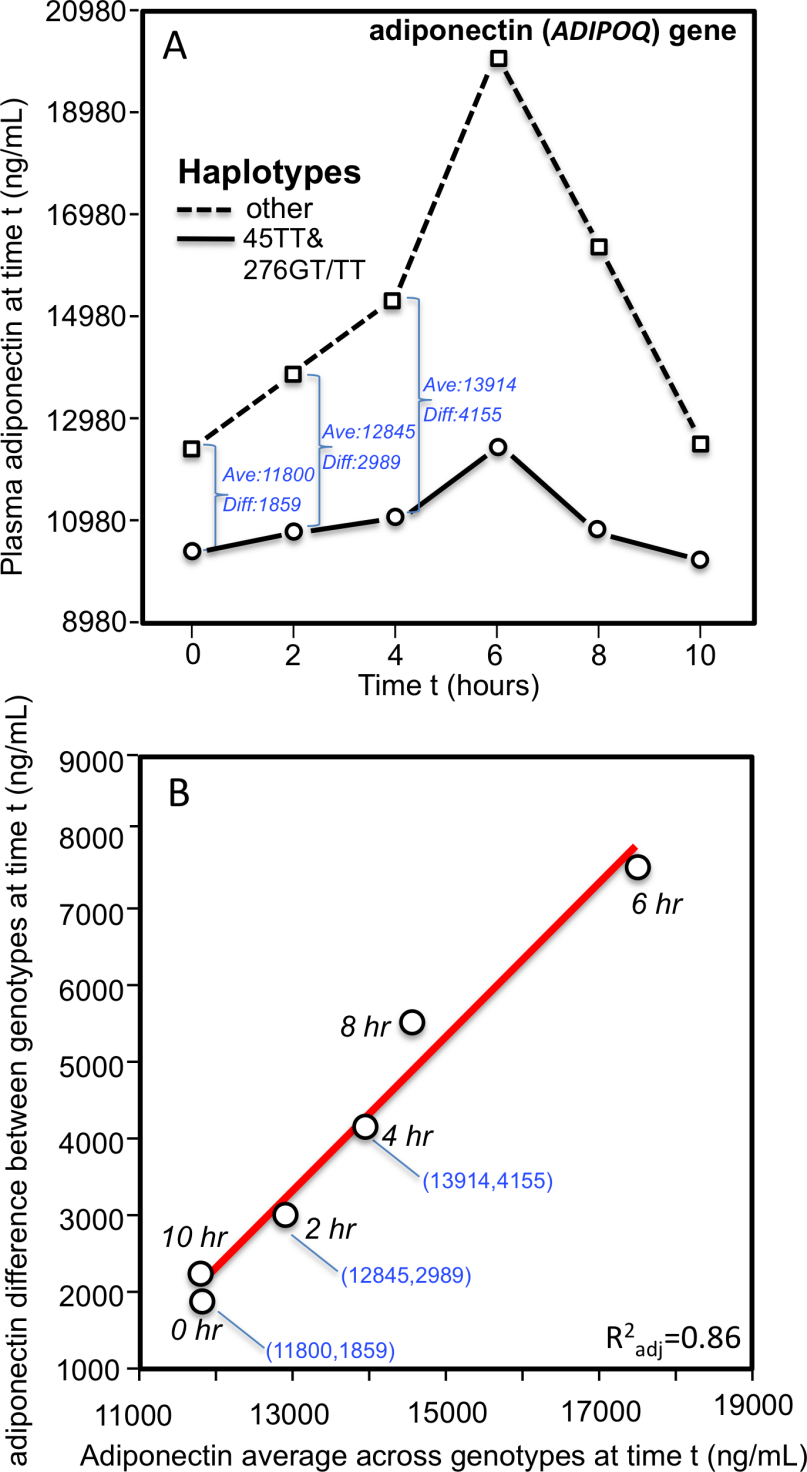
Adiponectin response to an oral fat tolerance test by 45TT (rs2241766) and 276GT/TT (rs1501299) *ADIPOQ* haplotypes. (A) Re-rendering of Musso et al.’s 2008 published adiponectin response to an oral fat tolerance test by 45TT (rs2241766) and 276GT/TT (rs1501299) *ADIPOQ* haplotypes (B) regression plot showing the genotypes difference (dependent variable) increased linearly with increasing adiponectin concentrations (independent variable). The genotype-specific mean concentrations were extracted from their figure 1C using the Microsoft PowerPoint formatting palette.

### Caveats and limitations

None of the SNPs identified to date explain any more than a few percent of adiponectin heritability, which means that the effects of any particular SNP is not necessarily constrained by the results of Fig. 1. Exceptions to Fig. 1 include Hara et al. (2002) report of significant adiponectin differences between *ADIPOQ* rs1501299 genotypes for obese Japanese whose mean concentrations were low, but not lean Japanese whose mean adiponectin concentrations were higher; and Gupta et al. reported that the *ADIPOQ* rs2241766 polymorphism significantly affected adiponectin in patients with nonalcoholic fatty liver disease but not controls despite the lower mean concentration of the patients (4.8 vs. 7.2 µg/ml) (Gupta et al., 2012). We also acknowledge that the simple estimates of *h*^2^ from Falconer’s formula probably do not adequately describe adiponectin inheritance (Falconer & Mackay, 1996), i.e., those derived from *β*_OP_ may include shared environmental effects, and those derived from *β*_FS_ may include shared environment and dominance effects and unmet restrictions on assortative mating. We note that the analyses were based on total rather than the biologically more active high molecular weight adiponectin. Finally, quantile-dependent expressivity represents an alternative interpretation to the gene-environment and precision medicine interpretations presented by others, but our analyses do not negate the original interpretation. Our analyses do not address the relationships of adiponectin to disease risk factors or endpoints, and therefore cannot provide insight to adiponectin paradox regarding all-cause and cardiovascular mortality (Menzaghi & Trischitta, 2018).

In conclusion, heritability of adiponectin concentrations is quantile-dependent, which appears to explain the stronger heritability in women in accordance with their higher concentrations, and is consistent with the interactions of genes with thiazolidinedione, adiposity, and postprandial changes reported by others. Prior reports of adiponectin heritability overlooked the effects of sex on heritability because of their use parametric statistics requiring logarithmic transformations. Genome-wide association studies of adiponectin also exclusively report on logarithmically transformed concentrations. Should we have chosen to log-transform adiponectin concentrations, the analyses would still have shown quantile-specific effects, but with heritability decreasing with increasing concentrations (Fig. S1). We analyzed untransformed adiponectin concentrations because quantile-regression does not require normality, and no biological rationale has been proposed for their logarithmic transformation. Parenthetically, the significant interactions reported by Kang et al. (2005), De Luis et al. (2020), De Luis et al. (2018), De Luis et al. (2019), Divella et al. (2017), Aller et al. (2019) and Garcia-Garcia et al. (2014) were all based on untransformed adiponectin concentrations.

##  Supplemental Information

10.7717/peerj.10099/supp-1 Supplemental Information 1 Quantile-specific heritability of log-transformed adiponectin concentrations

## Abbreviations

ADIPOQ: Adiponectin, C1Q And Collagen Domain Containing
APOA5: Apolipoprotein A5 gene
*β*_FS_: Full-sib regression slope
*β*_OM_: Offspring mid-parental regression slope
*β*_OP_: Offspring-parent regression slope
BMI: Body mass index
ELISA: Enzyme-linked immunosorbent assay
GWAS: Genome-wide association studies
h^2^: Heritability in the narrow sense
NHLBI: National Heart Lung and Blood Institute
Q-Q: plot Quantile-quantile plot
SD: Standard deviation
SE: Standard error
SNP: Single nucleotide polymorphism
T2DM: Type 2 diabetes mellitus

## Additional Information and Declarations

### Data Availability

The following information was supplied regarding data availability: The data are not being published in accordance with the data use agreement between the NIH National Heart Lung, and Blood Institute and Lawrence Berkeley National Laboratory. However, the data that support the findings of this study are available from NIH National Heart Lung, and Blood Institute Biologic Specimen and Data Repository Information Coordinating Center directly through the website https://biolincc.nhlbi.nih.gov/my/submitted/request/. (National Heart, Lung, and Blood Institute, 2020b) Restrictions apply to the availability of these data, which were used under license for this study. For access to the data, please contact the Blood Institute Biologic Specimen and Data Repository Information Coordinating Center to find information on the human use approval and data use agreement requiring signature by an official with signing authority for their institute. The public summary-level phenotype data may be browsed at the dbGaP study home page (Genotypes and Phenotypes, 2020a).

## References

[ref-1] Al-Daghri NM, Al-Attas OS, Alokail MS, Alkharfy KM, Yakout SM, Sabico SB, Gibson GC, Chrousos GP, Kumar S (2011). Parent-offspring transmission of adipocytokine levels and their associations with metabolic traits. PLOS ONE.

[ref-2] Aller R, Izaola O, Primo D, De Luis DA (2019). The effect of single-nucleotide polymorphisms at the ADIPOQ gene locus rs1501299 on metabolic parameters after 9 mo of a high-protein/low-carbohydrate versus a standard hypocaloric diet. Nutrition.

[ref-3] Berra M, Armillotta F, D’Emidio L (2006). Testosterone decreases adiponectin levels in female to male transsexuals. Asian J Androl.

[ref-4] Berthier MT, Houde A, Côté M (2005). Impact of adiponectin gene polymorphisms on plasma lipoprotein and adiponectin concentrations of viscerally obese men. Journal of Lipid Research.

[ref-5] Bouatia-Naji N, Meyre D, Lobbens S, Séron K, Fumeron F, Balkau B (2006). ACDC/adiponectin polymorphisms are associated with severe childhood and adult obesity. Diabetes.

[ref-6] Butte NF, Comuzzie AG, Cai G, Cole SA, Mehta NR, Bacino CA (2005). Genetic and environmental factors influencing fasting serum adiponectin in Hispanic children. Journal of Clinical Endocrinology and Metabolism.

[ref-7] Cesari M, Narkiewicz K, De Toni R, Aldighieri E, Williams CJ, Rossi GP (2007). Heritability of plasma adiponectin levels and body mass index in twins. Journal of Clinical Endocrinology and Metabolism.

[ref-8] Chuang LM, Chiu YF, Sheu WH, Hung YJ, Ho LT, Grove J, Rodriguez B, Quertermous T, Chen YD, Hsiung CA, Tai TY, Stanford Asia-Pacific Program of Hypertension and Insulin Resistance Study Group (2004). Biethnic comparisons of autosomal genomic scan for loci linked to plasma adiponectin in populations of Chinese and Japanese origin. Journal of Clinical Endocrinology and Metabolism.

[ref-9] Comuzzie AG, Funahashi T, Sonnenberg G, Martin LJ, Jacob HJ, Black AE, Maas D, Takahashi M, Kihara S, Tanaka S, Matsuzawa Y, Blangero J, Cohen D, Kissebah A (2001). The genetic basis of plasma variation in adiponectin, a global endophenotype for obesity and the metabolic syndrome. Journal of Clinical Endocrinology and Metabolism.

[ref-10] Corbi G, Polito R, Monaco ML, Cacciatore F, Scioli M, Ferrara N, Nigro EDanieleA (2019). Adiponectin expression and genotypes in Italian people with severe obesity undergone a hypocaloric diet and physical exercise program. Nutrients.

[ref-11] Dastani Z, Hivert MF, Timpson N, Perry JR, Yuan X, Scott RA, Henneman P, Heid IM, Kizer JR, Lyytikainen LP (2012). Novel loci for adiponectin levels and their influence on type 2 diabetes and metabolic traits: a multi- ethnic meta-analysis of 45, 891 individuals. PLOS Genetics.

[ref-12] Dawber TR, Meadors GF, Moore FEJ (1951). Epidemiological approaches to heart disease: the Framingham Study. American Journal of Public Health.

[ref-13] De Luis DA, Calvo SG, Pacheco D, Ovalle HF, Aller R (2018). Adiponectin gene variant rs266729: relation to lipid profile changes and circulating adiponectin after bariatric surgery. Surgery for Obesity and Related Diseases.

[ref-14] De Luis DA, Primo D, Izaola O, Aller R (2020). Adiponectin gene variant rs266729 interacts with different macronutrient distribution of two different hypocaloric diets. Lifestyle Genomics.

[ref-15] De Luis DA, Primo D, Izaola O, Gomez Hoyos E, Lopez Gomez JJ, Ortola A (2019). Role of the variant in adiponectin gene rs266729 on weight loss and cardiovascular risk factors after a hypocaloric diet with the Mediterranean pattern. Nutrition.

[ref-16] Divella R, Daniele A, Mazzocca A, Abbate I, Casamassima P, Caliandro C, Ruggeri E, Naglieri E, Sabbà C, De Luca R (2017). ADIPOQ rs266729 G/C gene polymorphism and plasmatic adipocytokines connect metabolic syndrome to colorectal cancer. Journal of Cancer.

[ref-17] Dosaev T, Prakash J, Livshits G (2014). Contribution of body composition components and soft-tissue biochemical factors to genetic variation of body mass index (BMI) in an ethnically homogeneous population. American Journal of Human Biology.

[ref-18] Falconer DS, Mackay TFC (1996). Introduction to quantitative genetics.

[ref-19] Garcia-Garcia MR, Morales-Lanuza MA, Campos-Perez WY, Ruiz-Madrigal B, Maldonado-Gonzalez M, Vizmanos B, Hernandez-Cañaveral I, Yañez Sanchez I, Roman S, Panduro A, Martinez-Lopez E (2014). Effect of the ADIPOQ gene -11391G/A polymorphism is modulated by lifestyle factors in Mexican subjects. Journal of Nutrigenetics and Nutrigenomics.

[ref-20] Genotypes and Phenotypes (dbGaP) (2020a). Framingham cohort. dbGaP Study Accession:phs000007.v30.p11. https://www.ncbi.nlm.nih.gov/projects/gap/cgi-bin/study.cgi?study_id=phs000007.v30.p11.

[ref-21] Gould WW (1992). Quantile regression with bootstrapped standard errors. Stata Technical Bulletin.

[ref-22] Gu HF (2009). Biomarkers of adiponectin: plasma protein variation and genomic DNA polymorphisms. Biomark Insights.

[ref-23] Guo X, Saad MF, Langefeld CD, Williams AH, Cui J, Taylor KD, Norris JM, Jinagouda S, Darwin CH, Mitchell BD, Bergman RN, Sutton B, Chen YD, Wagenknecht LE, Bowden DW, Rotter JI (2006). Genome-wide linkage of plasma adiponectin reveals a major locus on chromosome 3q distinct from the adiponectin structural gene: the IRAS family study. Diabetes.

[ref-24] Gupta AC, Misra R, Sakhuja P, Singh Y, Basir SF, Sarin SK (2012). Association of adiponectin gene functional polymorphisms (-11377C/G and +45T/G) with nonalcoholic fatty liver disease. Gene.

[ref-25] Hara K, Boutin P, Mori Y, Tobe K, Dina C, Yasuda K, Yamauchi T, Otabe S, Okada T, Eto K, Kadowaki H, Hagura R, Akanuma Y, Yazaki Y, Nagai R, Taniyama M, Matsubara K, Yoda M, Nakano Y, Tomita M, Kimura S, Ito C, Froguel P, Kadowaki T (2002). Genetic variation in the gene encoding adiponectin is associated with an increased risk of type 2 diabetes in the Japanese population. Diabetes.

[ref-26] Heid IM, Henneman P, Hicks A, Coassin S, Winkler T (2010). Clear detection of ADIPOQ locus as the major gene for plasma adiponectin: results of genome-wide association analyses including 4659 European individuals. Atherosclerosis.

[ref-27] Henneman P, Aulchenko YS, Frants RR, Zorkoltseva IV, Zillikens MC, Frolich M, Oostra BA, Van Dijk KW, Van Duijn CM (2010). Genetic architecture of plasma adiponectin overlaps with the genetics of metabolic syndrome-related traits. Diabetes Care.

[ref-28] Hicks C, Zhu X, Luke A, Kan D, Adeyemo A, Wu X, Cooper RS (2007). A genome-wide scan of loci linked to serum adiponectin in two populations of African descent. Obesity.

[ref-29] Jee SH, Sull JW, Lee JE, Shin C, Park J (2010). Adiponectin concentrations: a genome-wide association study. American Journal of Human Genetics.

[ref-30] Kang ES, Park SY, Kim HJ, Ahn CW, Nam M, Cha BS, Lim SK, Kim KR, Lee HC (2005). The influence of adiponectin gene polymorphism on the rosiglitazone response in patients with type 2 diabetes. Diabetes Care.

[ref-31] Kannel WB, Feinleib M, McNamara PM, Garrison RJ, Castelli WP (1979). An investigation of coronary heart disease in families. The Framingham offspring study. American Journal of Epidemiology.

[ref-32] Karlin S, Cameron EC, Williams PT (1981). Sibling and parent–offspring correlation estimation with variable family size. Proceedings of the National Academy of Sciences of the United States of America.

[ref-33] Koenker R, Hallock KF (2001). Quantile regression. Journal of Economic Perspectives.

[ref-34] Li S, Shin HJ, Ding EL, Van Dam RM (2009). Adiponectin levels and risk of type 2 diabetes: a systematic review and meta-analysis. JAMA.

[ref-35] Lindsay RS, Funahashi T, Krakoff J, Matsuzawa Y, Tanaka S, Kobes S, Bennett PH, Tataranni PA, Knowler WC, Hanson RL (2003). Genome-wide linkage analysis of serum adiponectin in the Pima Indian population. Diabetes.

[ref-36] Ling H, Waterworth DM, Stirnadel HA, Pollin TI, Barter PJ (2009). Genome-wide linkage and association analyses to identify genes influencing adiponectin levels: the GEMS study. Obesity.

[ref-37] Liu PH, Jiang YD, Chen WJ, Chang CC, Lee TC, Sun HS, Chuang LM (2008). Genetic and environmental influences on adiponectin, leptin, and BMI among adolescents in Taiwan: a multivariate twin/sibling analysis. Twin Research and Human Genetics.

[ref-38] Menzaghi C, Salvemini L, Paroni G, DeBonis C, Mangiacotti D, Fini G, Doria A, DiPaola R, Trischitta V (2010). Circulating high molecular weight adiponectin isoform is heritable and shares a common genetic background with insulin resistance in nondiabetic White Caucasians from Italy: evidence from a family-based study. Journal of Internal Medicine.

[ref-39] Menzaghi C, Trischitta V (2018). The adiponectin paradox for all-Ccause and cardiovascular mortality. Diabetes.

[ref-40] Musso G, Gambino R, De Michieli F, Durazzo M, Pagano G, Cassader M (2008). Adiponectin gene polymorphisms modulate acute adiponectin response to dietary fat: possible pathogenetic role in NASH. Hepatology.

[ref-41] National Heart, Lung, and Blood Institute (2020b). NIH National Heart Lung and Blood Institute Biologic Specimen and Data Repository Information Coordinating Center. https://biolincc.nhlbi.nih.gov/home/.

[ref-42] Pollin TI, Tanner K, O’connell JR, Ott SH, Damcott CM, Shuldiner AR, McLenithan JC, Mitchell BD (2005). Linkage of plasma adiponectin levels to 3q27 explained by association with variation in the APM1 gene. Diabetes.

[ref-43] Richards JB, Waterworth D, O’Rahilly S, Hivert MF, Loos RJ (2009). A genome-wide association study reveals variants in ARL15 that influence adiponectin levels. PLOS Genetics.

[ref-44] Riestra P, Gebreab SY, Xu R, Khan RJ, Bidulescu A, Correa A, Tekola-Ayele F, Davis SK (2015). Gender-specific associations between ADIPOQ gene polymorphisms andadiponectin levels and obesity in the Jackson Heart Study cohort. BMC Medical Genetics.

[ref-45] Splansky GL, Corey D, Yang Q, Atwood LD, Cupples LA, Benjamin EJ, D’Agostino Sr RB, Fox CS, Larson MG, Murabito JM, O’Donnell CJ, Vasan RS, Wolf PA, Levy D (2007). The third generation cohort of the national heart, lung, and blood institute’s Framingham heart study: design, recruitment, and initial examination. American Journal of Epidemiology.

[ref-46] Swarbrick MM, Havel PJ (2008). Physiological, pharmacological, and nutritional regulation of circulating adiponectin concentrations in humans. Metabolic Syndrome and Related Disorders.

[ref-47] Vasseur F, Helbecque N, Dina C, Lobbens S, Delannoy V, Gaget S, Boutin P, Vaxillaire M, Leprêtre F, Dupont S, Hara K, Clément K, Bihain B, Kadowaki T, Froguel P (2002). Single-nucleotide polymorphism haplotypes in the both proximal promoter and exon 3 of the APM1 gene modulate adipocyte-secreted adiponectin hormone levels and contribute to the genetic risk for type 2 diabetes in French Caucasians. Human Molecular Genetics.

[ref-48] Vaughan LK, Wiener HW, Aslibekyan S, Allison DB, Havel PJ, Stanhope KL, O’Brien DM, Hopkins SE, Lemas DJ, Boyer BB, Tiwari HK (2015). Linkage and association analysis of obesity traits reveals novel loci and interactions with dietary n-3 fatty acids in an Alaska Native (Yup’ik) population. Metabolism: Clinical and Experimental.

[ref-49] Wilk MB, Gnanadesikan R (1968). Probability plotting methods for the analysis of data. Biometrika.

[ref-50] Williams PT (2012). Quantile-specific penetrance of genes affecting lipoproteins, adiposity and height. PLOS ONE.

[ref-51] Williams PT (2020a). Gene-environment interactions due to quantile-specific heritability of triglyceride and VLDL concentrations. Scientic Reports.

[ref-52] Williams PT (2020b). Quantile-dependent expressivity of postprandial lipemia. PLOS ONE.

[ref-53] Williams PT (2020c). Quantile-dependent heritability of computed tomography, dual-energy X-ray absorptiometry, anthropometric, and bioelectrical measures of adiposity. International Journal of Obesity.

[ref-54] Williams PT (2020d). Quantile-specific heritability may account for gene-environment interactions involving coffee consumption. Behavior Genetics.

[ref-55] Williams PT (2020e). Quantile-specific heritability of high-density lipoproteins with implications for precision medicine. Journal of Clinical Lipidology.

[ref-56] Williams PT (2020f). Quantile-specific heritability of intakes of alcohol but not other macronutrients. Behavior Genetics.

[ref-57] Williams PT (2020g). Spirometric traits show quantile-dependent heritability, which may contribute to their gene-environment interactions with smoking and pollution. PeerJ.

[ref-58] Winer BJ, Brown DR, Michels KM (1991). Statistical principles in experimental design.

[ref-59] Zachariah JP, Quiroz R, Nelson KP, Teng Z, Keaney JF, Sullivan LM, Vasan RS (2017). Prospective relation of circulating adipokines to incident metabolic syndrome: the Framingham Heart Study. Journal of the American Heart Association.

