# Supplementary figures and images for: Quantile-dependent expressivity of plasma adiponectin concentrations may explain its sex-specific heritability, gene-environment interactions, and genotype-specific response to postprandial lipemia
Source: PeerJ. 2020 Oct 14;8:e10099. doi: 10.7717/peerj.10099 (PMC7568478; doi:10.7717/peerj.10099)

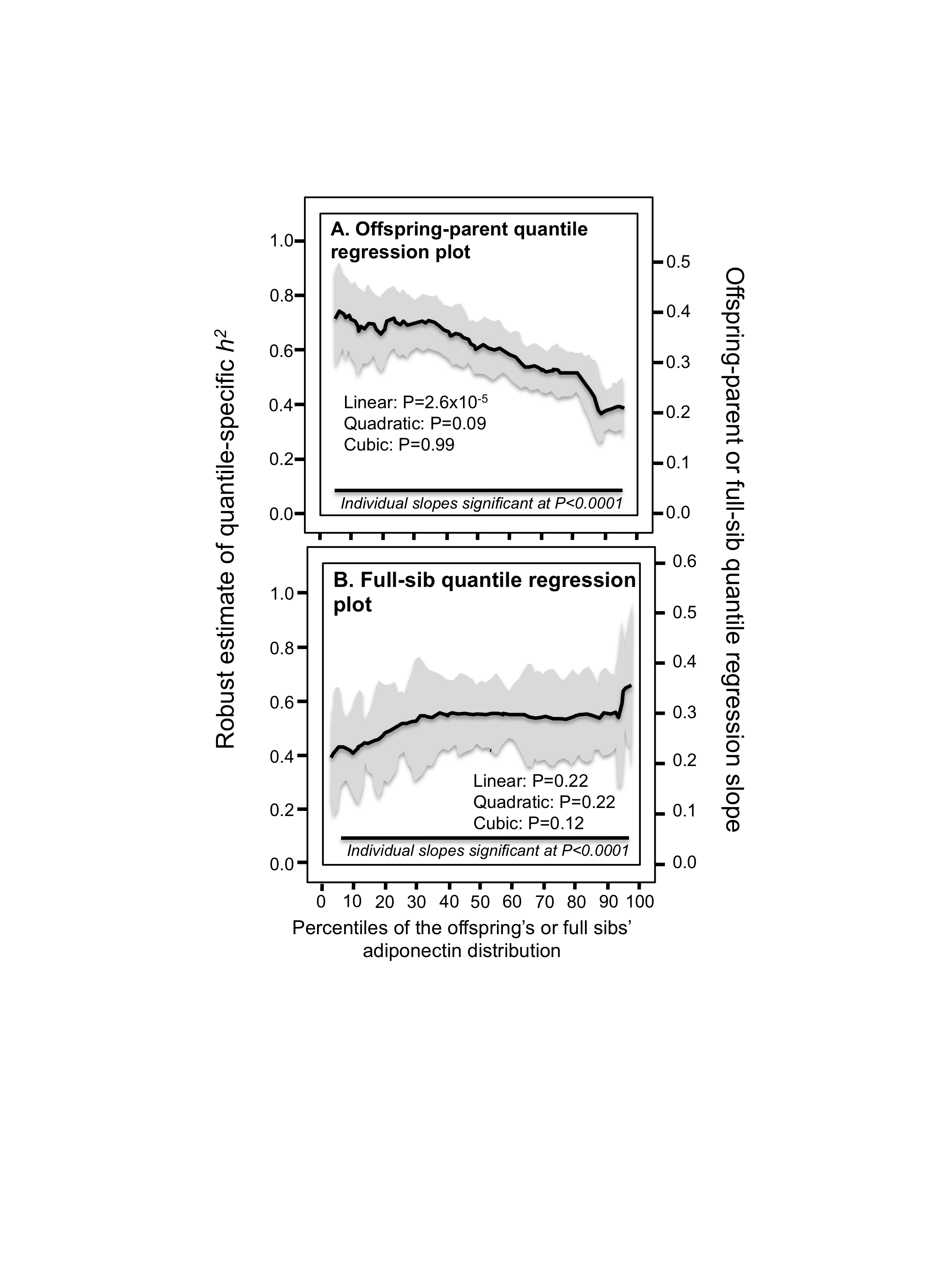

Supplement: Supplemental Information 1 [file peerj-08-10099-s001.png]
